# Live-cell monitoring of protein localization to membrane rafts using protein-fragment complementation

**DOI:** 10.1042/BSR20191290

**Published:** 2020-01-03

**Authors:** Maria Merezhko, Emmi Pakarinen, Riikka-Liisa Uronen, Henri J. Huttunen

**Affiliations:** 1 Neuroscience Center, HiLIFE, University of Helsinki, 00014 Helsinki, Finland; 2 Institute of Biotechnology, HiLIFE, University of Helsinki, 00014 Helsinki, Finland

**Keywords:** high-throughput screening, lipid microdomains, lipid rafts, protein-fragment complementation assay

## Abstract

The plasma membrane consists of a variety of discrete domains differing from the surrounding membrane in composition and properties. Selective partitioning of protein to these microdomains is essential for membrane functioning and integrity. Studying the nanoscale size and dynamic nature of the membrane microdomains requires advanced imaging approaches with a high spatiotemporal resolution and, consequently, expensive and specialized equipment, unavailable for most researchers and unsuited for large-scale studies. Thus, understanding of protein partitioning to the membrane microdomains in health and disease is still hampered by the lack of inexpensive live-cell approaches with an appropriate spatial resolution. Here, we have developed a novel approach based on *Gaussia princeps* luciferase protein-fragment complementation assay to quantitively investigate protein partitioning to cholesterol and sphingomyelin-rich domains, sometimes called ‘lipid rafts’, in intact living cells with a high-spatial resolution. In the assay, the reporter construct, carrying one half of the luciferase protein, is targeted to lipid microdomains through the fused acetylation motif from Src-family kinase Fyn. A protein of interest carries the second half of the luciferase protein. Together, this serves as a reversible real-time sensor of raft recruitment for the studied protein. We demonstrated that the assay can efficiently detect the dynamic alterations in raft localization of two disease-associated proteins: Akt and APP. Importantly, this method can be used in high-throughput screenings and other large-scale studies in living cells. This inexpensive, and easy to implement raft localization assay will benefit all researchers interested in protein partitioning in rafts.

## Introduction

Cellular membranes comprise hundreds of different lipid species and at least as many different proteins. These components are in constant dynamic interaction, attracting and repelling one another [1,2]. Some membrane components, such as cholesterol and sphingomyelin attract each other, segregating laterally to form domains whose lipid and protein composition differs from the rest of the membrane [1]. These domains, initially called ‘lipid rafts’, are more ordered and tightly packed than the surrounding membrane [1]. They are thought to occur in the nanometer scale and are not restricted to cholesterol and sphingomyelin-based domains but are also formed by e.g. ceramide and phosphatidyl inositol 4-5-bisphosphate (PIP_2_) [3,4]. Proteins preferentially partition to different membrane domains depending on their properties; for instance, glycophosphatidylinositol (GPI)-anchored proteins in the outer leaflet and acylated proteins in the inner leaflet of the lipid bilayer have a preference to the cholesterol/sphingomyelin-rich domains or ‘rafts’ [5].

Methodological limitations in the studies of the lipid domains are set by their nanoscale size and transient dynamic nature. Thus, research approaches with high spatiotemporal resolution are required for the investigation of lipid domains in the plasma membrane. The emergence of super-resolution optical microscopy techniques, such as photoactivated localization microscopy (PALM), stimulated emission depletion (STED), structured illumination microscopy (SIM) or near-field scanning optical microscopy (NSOM), has helped to investigate dynamic partitioning of proteins to lipid microdomains at scale of up to ∼20 nm [6–8]. Although the super-resolution approaches based on the patterned light illumination (such as STED and SIM) have allowed observation in millisecond timescale, the alternative approaches such as single particle tracking (SPT) or fluorescence correlation spectroscopy (FCS) are able to provide considerably superior dynamic measures with a time resolution of microseconds [7–10]. Moreover, the combination and modifications of these tools have further increased their sensitivity, allowing to combine both high spatial and high temporal resolution in a single technique such as STED-FCS or SPT based on interferometric scattering microscopy (iSCAT) [7].

The appropriate methods for studies of membrane microdomain composition and dynamics, however, involve complex equipment and/or data analysis and are rather laborious and expensive. As a consequence, more simplistic but suboptimal approaches are still extensively utilized especially for the initial exploration of membrane raft partitioning of proteins to the cholesterol/sphingomyelin-rich domains. A widely used approach in this category employs density gradient fractionation using either non-ionic detergent-based or detergent-free methods that allow to separate the cellular membrane into detergent-soluble membrane fractions (DSMs) and detergent-resistant membrane fractions (DRMs) [11]. DRMs are enriched in cholesterol and sphingomyelin and contain a subset of associated proteins, which led to the highly debated assumption that DRMs correspond to cholesterol/sphingomyelin-rich microdomains [1,12,13]. However, it is clear now that this approach has many significant limitations, but most importantly, it breaks the membrane and destroys cell morphology and therefore cannot reflect the native content of the transient nanoscale lipid domains in living cells [14,15].

Another simplistic approach is the application of conventional, instead of high-resolution, microscopy to visualize the colocalization of proteins with putative lipid microdomain probe (such as fluorophore-conjugated CTxB, the membrane-binding subunit of cholera toxin that binds GM1 gangliosides in lipid microdomains) to draw conclusions about protein localization within the plasma membrane [16]. The resolution of conventional microscopy is, however, insufficient to make reliable conclusions about the content of the nanoscale structures. Moreover, many of the probes selective to membrane microdomains have limited specificity and may rearrange lipid rafts, thus introducing artifacts [7,17]. Thus, there is a clear need for the development of simple and high-throughput approaches for the initial research and pharmacological studies, which could measure partitioning of proteins to cholesterol/sphingomyelin-rich domains in living cells. These methods should not be seen as a substitute for imaging approaches with high spatiotemporal resolution, but answer more simplistic questions.

To overcome the limitations of the existing methods, we have developed an assay based on the *Gaussia princeps* luciferase Protein-fragment Complementation Assay (PCA) to study localization of proteins to cholesterol-based membrane domains in intact live cells [18]. In this assay, the reporter construct carrying one half of the luciferase protein (either N-terminal 93 amino-acid fragment or C-terminal 76 amino-acid fragment) fused to the 10 amino acid long acetylation motif from the Src-family kinase Fyn serves as a reversible real-time sensor of raft recruitment for a protein carrying the complementary half of the luciferase protein [19,20]. This strategy has allowed us to develop a high-throughput sensitive live-cell approach, which not only allows to detect the membrane raft localization of a protein of interest but also allows application of chemical biology methods to modulate and dissect the mechanisms of this localization. The assay does not involve rare or expensive equipment or software for the data analysis, but provides good temporal resolution, requires little starting material, is low cost and easy to implement.

## Materials and methods

### Plasmid constructs and chemicals

The original split *G. princeps* luciferase (GLuc) plasmids were donated by Dr Stephen Michnick (Université de Montréal, Montreal, Canada); the plasmids were constructed in the pcDNA3.1/zeo (Invitrogen) backbone. The GLuc1/2 constructs were further modified by fusing the HA-tag (residues 98–106 from human influenza hemagglutinin) to the N-terminus of GLuc to facilitate the immunodetection; HA-tag sequence was amplified from pEAK12-ADAM10/HA plasmid (a kind gift from Dr Stephan Lichtenthaler, Ludwig-Maximilians-Universität München, Germany) [21]. LR sequence (the N-terminal 10 amino acids from Fyn kinase) was amplified from Fyn cDNA (GenBank accession number: BC032496). LR(C3,6S)-GLuc1/HA and LR(G2A)-GLuc1/HA constructs were generated with PCR-based site-directed mutagenesis through amplifying LR-GLuc1 sequence with the following primers: 5′-TATGGATCCACCGCCATGGGCTCTGTGCAATCTAAGGAT-3′ (forward primer for LR(C3,6S)-GLuc1/HA); 5′-TATGGATCCACCGCCATGGCCTGTGTGCAATGTAAGGAT-3′ (forward primer for LR(G2A)-GLuc1/HA), and 5′-CTCTAGATTAGCCTATGCCGCCCTGTGCGG-3′ (reverse primer for both constructs). PCRs were performed using Phusion high-fidelity DNA polymerase and LR-GLuc1/HA construct as the template; the amplified fragments were cloned into the GLuc1/HA vector. The GLuc-tagged Amyloid precursor protein APP_695_ (neuronal isoform lacking the KPI domain) construct (APP-GLuc2) was generated and donated by Dr Oksana Berezovska (Massachusetts General Hospital, Boston, MA). All other APP constructs used in the present study (β-secretase cleaved C-terminal fragment of APP (APP-βCTF)-GLuc2 and APP Intracellular Domain (APP-AICD)-GLuc2) were cloned based on GLuc-APP. The cDNA of β-secretase1 (BACE1; GeneBank accession number: NM_012104.4) was donated by Dr Dora Kovacs (Massachusetts General Hospital, Boston, MA). The cDNAs for Fyn and Akt (GeneBank accession number: BC000479) were produced synthetically (GeneArt, Thermo Fisher Scientific). For all PCA constructs used in the present study, the GLuc fragment was placed in the cytosolic C-terminus after a (GGGGS)_2_SG linker. The identity of all constructs was confirmed by DNA sequencing.

Methyl-β-cyclodextrin (mβCD) and cholesterol were purchased from Sigma-Aldrich. Human insulin was purchased from Novo Nordisk.

### Cell culture and transfection

Neuro-2A (N2A) mouse neuroblastoma cells (ATCC) were maintained in Dulbecco’s Modified Eagle Medium (DMEM, Corning) supplemented with 10% (v/v) of fetal bovine serum (Invitrogen) and 1% (v/v) streptomycin, penicillin and L-glutamine (Lonza) at 37°C in a water-saturated air, 5% CO_2_ atmosphere. Transfection of N2A cells was performed 24 h after plating using JetPEI reagent (Polyplus) according to manufacturer’s instructions. The transfection conditions were optimized to reach at least 80% transfection efficiency.

### Western blotting

**Figure 1:**
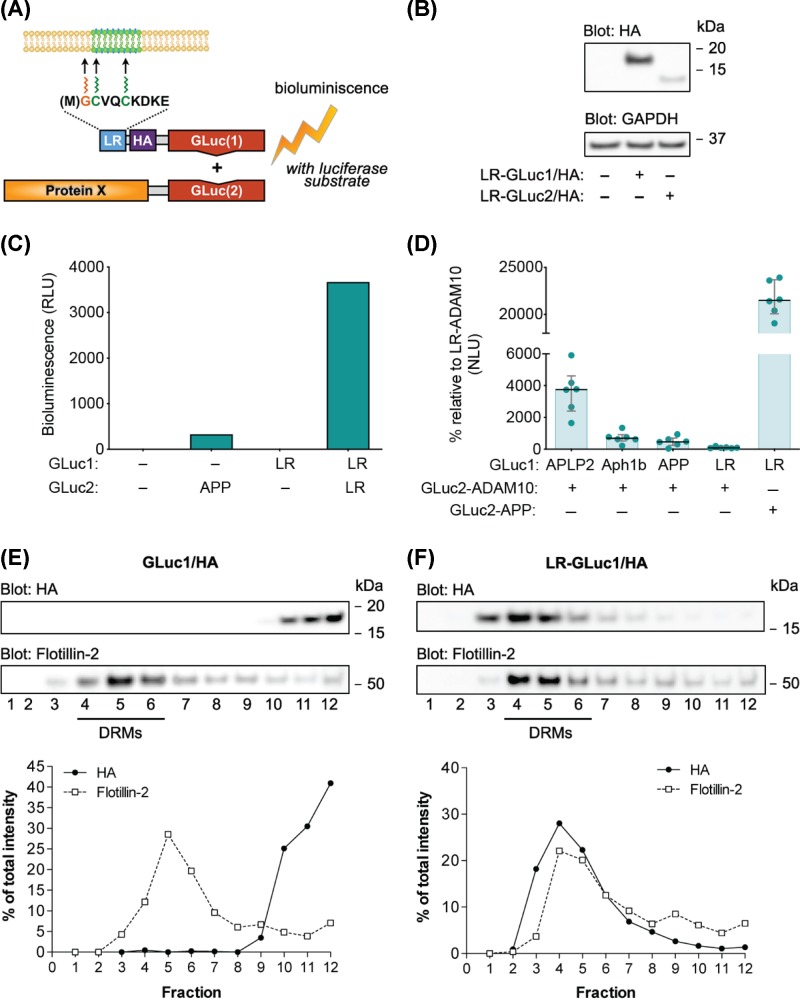
The design and validation of a live-cell membrane raft-localization reporter assay (LR-PCA) (**A**) Graphical presentation of LR-PCA reporter constructs and the assay principle. The N-terminal 10 amino acids from Fyn kinase are linked to HA-tag and GLuc fragment via a (GGGGS)_2_SG linker. Met1 is removed during translation. Myristoylation of the Gly2 drives the reporter to the membrane, and acylation of Cys3 and Cys6 recruits it to lipid rafts; GLuc1 and GLuc2 – 5′ and 3′ fragment of codon-optimized for mammalian expression form of *G. princeps* luciferase; HA - hemagglutinin tag from human influenza; LR – lipid raft targeting motif from Fyn kinase. (**B**) Expression levels of LR-GLuc1/HA and LR-GLuc2/HA reporter constructs in N2A cells. Cells were transiently transfected with indicated constructs; proteins were extracted 48 h post-transfection. Cell extracts were analyzed on Western blots with HA antibody, with the GAPDH antibody used as a loading control. Reporters migrated differently in SDS-PAGE due to the size of their GLuc-fragment (GLuc1 10.1 kDa and GLuc2 8.2 kDa). (**C**) Validation of LR-PCA for detection specificity. N2A cells were transiently transfected with indicated constructs; luminescence signal was measured 48 h post-transfection in live cells. Little or no luminescence is detected when one or both co-expressed plasmids were GLuc1/2 expressing the indicated GLuc fragment alone. The values are bioluminescence signals recorded from expressed pairs of reporter constructs. The panel shows the results of the representative experiment since it is impossible to average absolute bioluminescence values in PCA assay without a normalization due to the high variation of non-biological origin between replicas. Three additional replicas have shown a similar pattern (Supplementary Figure S1). (**D**) Validation of LR-PCA for detection specificity with a non-DRM protein. N2A cells were transiently transfected with indicated constructs; luminescence signal was measured 48 h post-transfection in live cells. Neglectable level of luminescence is detected when largely non-DRM ADAM10-GLuc2 was co-expressed with LR-GLuc1/HA, while the co-expression of ADAM10-GLuc2 with either APLP2-GLuc2, Aph1b-GLuc2 or APP-GLuc2, all known to interact with ADAM10, generated high-level of luminescence. When LR-GLuc1/HA was co expressed with the DRM protein APP, very high-level of luminescence was generated. The luciferase activity is expressed as normalized luminescence units (NLU). Each dot represents individual NLU value; *n* = 6. (**E** and **F**) Reporter validation by sucrose gradient fractionation: LR-GLuc1/HA reporter, but not empty GLuc1/HA, localizes to detergent-resistant membrane fractions (DRMs). N2A cells, transiently transfected with either empty GLuc1/HA or LR-GLuc1/HA, were solubilized in 0.5% (w/v) Lubrol and separated into DRM and non-DRM fractions by sucrose gradient density fractionation at 180,000 × *g* for 18 h at 4°C. After the run, fractions were collected from top to bottom, sonicated and analyzed by Western blotting. HA antibody was used to detect reporters, while Flotillin-2 antibody was used to identify DRM fractions. Relative optical intensities of HA and Flotillin-2 stainings, observed in Western blotting, were quantified and presented as a percentage per total staining intensity in all fractions.

**Figure 2:**
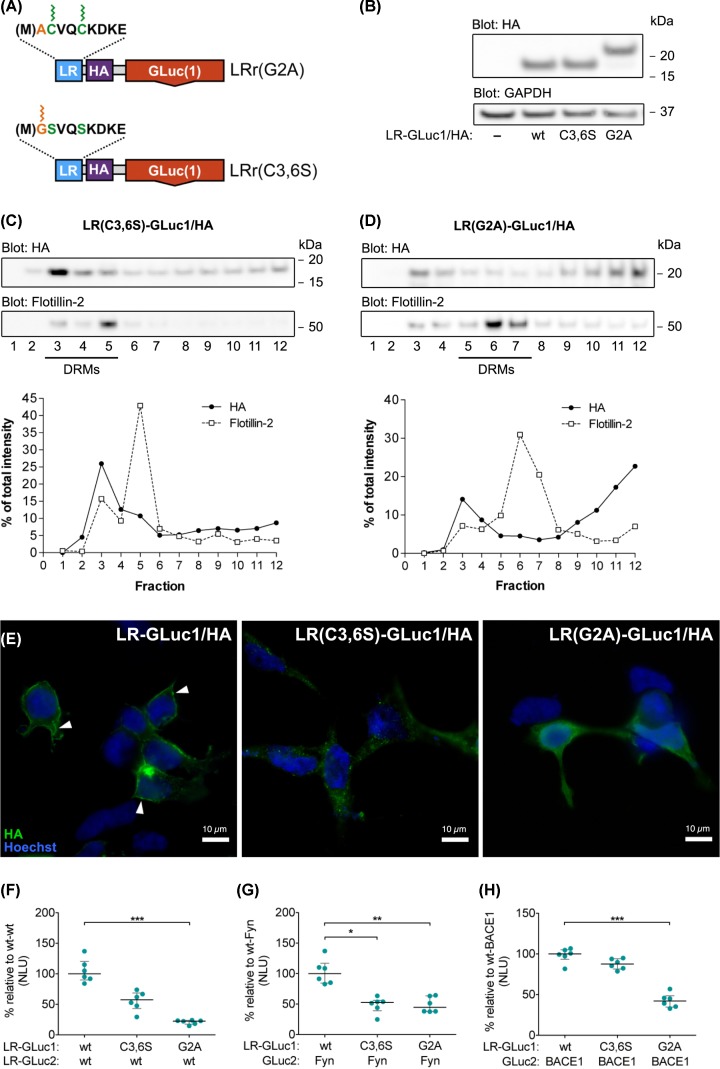
Acylation is necessary for raft localization of the LR-PCA reporters (**A**) Graphical presentation of acylation mutant LR-PCA reporter constructs showing mutated residues in LR-targeting signal; GLuc1 – 5′ fragment of humanized Gaussia luciferase; HA – hemagglutinin tag from human influenza; LR – lipid raft targeting motif from Fyn kinase. (**B**) Expression levels of LR-GLuc1/HA, LR(C3,6S)-GLuc1/HA and LR(G2A)-GLuc1/HA reporter constructs in N2A cells. Cells were transiently transfected with indicated constructs; proteins were extracted 48 h post-transfection. Cell extracts were analyzed on Western blots with HA antibody, with the GAPDH antibody used as a loading control. (**C** and **D**) Reporter localization by sucrose gradient fractionation: LR(C3,6S)-GLuc1/HA and LR(G2A)-GLuc1/HA reporters display different affinities to detergent-resistant membrane fractions (DRMs) compared with wild-type reporter. N2A cells, transiently transfected with either LR(C3,6S)-GLuc1/HA or LR(G2A)-GLuc1/HA, were subjected to fractionation and analysis as in Figure 1D–G. (**E**) Subcellular localization of LR-GLuc1/HA reporter and its fatty acylation mutants by immunofluorescence microscopy in N2A cells. Cells were transiently transfected with indicated constructs, fixed 24 h post-transfection and stained with HA antibody to visualize of LR-GLuc1/HA reporters; the nuclei were counterstained with Hoechst 33342. Arrowheads point to small membrane patches of LR-GLuc1/HA; scale bars = 10 μm. (**F–H**) The effect of fatty acylation site mutations on LR-GLuc1/HA reporter association with LR-GLuc2/HA, Fyn-GLuc2 and BACE1-GLuc2. N2A cells were transiently transfected with indicated constructs; luminescence signal was measured 48 h post-transfection in live cells. The luciferase activity is expressed as normalized luminescence units (NLU). Each dot represents individual NLU value, horizontal lines represent medians and IQR is shown in gray; in panels (H, I and J) *P* = 0.0005, *P* = 0.0033 and *P* = 0.0013, respectively; *n* = 6. Statistical significance was assessed using Kruskal–Wallis with Dunn’s post-hoc test; *P < 0.05, **P < 0.01, ***P < 0.001.

**Figure 3:**
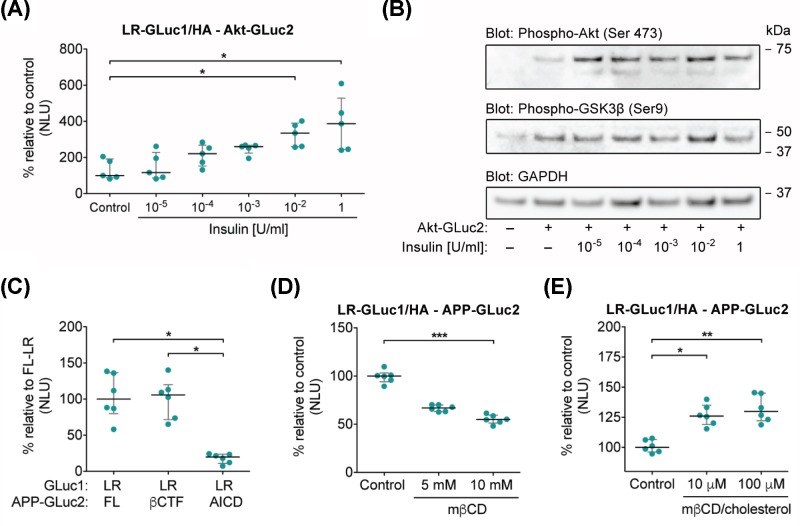
Dynamic localization of proteins to rafts (**A**) Functional validation of LR-PCA. LR-PCA demonstrates insulin-induced recruitment of Akt to rafts. N2A cells were transiently transfected with LR-GLuc1/HA and Akt-GLuc2, and treated with indicated concentrations of insulin for 1 h at 37°C before measurement of LR-PCA signals 48 h post-transfection in live cells; *P* = 0.0041; *n* = 5. (**B**) Insulin activates downstream phosphorylation of Akt and GSK3β in N2A cells. Cells were transiently transfected with Akt-GLuc2 and treated with indicated concentrations of insulin for 1 h prior to protein extraction (48 h post-transfection). Cell extracts were analyzed on Western blots with phospho-Akt(Ser473) and phospho-GSK3β(Ser9) antibodies, GAPDH antibody was used as a loading control. (**C**) Recruitment of APP and its proteolytic fragments to rafts as detected by LR-PCA. N2A cells were transiently transfected with indicated constructs; luminescence signal was measured 48 h post-transfection in live cells; *P* = 0.0034; *n* = 6. (**D**) Acute cholesterol depletion decreases the association of APP-GLuc2 with LR-GLuc1/HA reporter. N2A cells were transiently transfected with LR-GLuc1/HA and APP-GLuc2, treated with 5 or 10 mM of mβCD at 37°C for 30 min. LR-PCA signal was measured 48 h post-transfection in live cells; *P* = 0.0005; *n* = 6. (**E**) Acute cholesterol loading increases the association of APP-GLuc2 with LR-GLuc1/HA reporter. N2A cells were transiently transfected with LR-GLuc1/HA and APP-GLuc2, incubated in serum-free media for 9.5 h prior to exposure to 10 or 100 µM mβCD/cholesterol for 30 min. LR-PCA signal was measured 48 h post-transfection in live cells; *P* = 0.0029; *n* = 6. The luciferase activity is expressed as normalized luminescence units (NLU). Each dot represents individual NLU value, horizontal lines represent medians and IQR is shown in gray. Statistical significance was assessed using Kruskal–Wallis with Dunn’s post-hoc test. *P < 0.05, **P < 0.01 and ***P < 0.001.

The cells were plated on 6-well polystyrene plates and transfected with 3 μg of total DNA per well. Forty-eight hours after transfection, the cells were washed twice with ice-cold PBS, scraped and extracted on ice for 30 min in a buffer containing 10 mM Tris-HCl, pH 6.8, 1 mM EDTA, 150 mM NaCl, 1% (v/v) Triton X-100, 0.25% (v/v) Nonidet P-40, 1 μM NaF, protease and phosphatase inhibitor cocktail tablets (Roche Molecular Biochemicals). Cell debris was removed by centrifugation at 13,000 × *g*. Protein concentration of extracts was determined by BCA Protein Assay kit (Thermo Scientific), and equal amounts of total protein per sample were used. Protein samples were boiled at 80°C for 10 min in NuPAGE loading buffer (Invitrogen) and 0.25% (v/v) mercaptoethanol before loading on 4–12% Bis-Tris gel (Invitrogen). Electrophoresis was performed for 1 h using a constant voltage (160 V) in NuPAGE MES SDS running buffer (50 mM MES, 50 mM Tris Base, 0.1% (w/v) SDS, 1 mM EDTA, pH 7.3, Invitrogen) under reducing conditions using mercaptoethanol as reducing agents. Resolved proteins were transferred to a PVDF methanol-activated membrane (GE Healthcare) by semi-dry blotting (Bio-Rad) using Tris-glycine transfer buffer (25 mM Tris, 192 mM glycine, pH 8.3) with 20% (v/v) methanol. The PVDF membrane was directly blocked with a blocking buffer (5% (w/v) fat-free milk in Tris-buffered saline (TBS) with 0.05% Tween-20 (TBST)) for 40 min and then probed with primary antibodies. HA antibody (Sigma-Aldrich, 1:1000), GAPDH antibody (Millipore, 1:1000), Flotillin-2 antibody (Cell Signaling, 1:1000), Phospho-Akt(Ser473) antibody (Cell Signaling, 1:1000), Phospho-GSK3β(Ser9) antibody (Cell Signaling, 1:1000), horseradish peroxidase-conjugated secondary antibodies (1:6000) and ECL Western blotting detection reagent (Thermo Scientific) were used to detect chemiluminescence signal. Chemiluminescent signal was detected with the LAS-3000 imaging system (Fujifilm) and QuantityOne software (Bio-Rad) was used for semi-quantitative analysis of Western blots. Densiometric values of of HA, Phospho-Akt(Ser473) and Phospho- GSK3β(Ser9) staining in Figures 1B, 2B, and 3B were normalized to densiometric values of the corresponding GAPDH staining. The resulting values are normalized by the sum of measurements; in Figures 1B and 2B of all data points and expressed as % of the total staining, while in Figure 3 as % change to the control (the second column: + Akt-Gluc2/ - insulin).

### Protein-fragment complementation assay (PCA/LR-PCA)

The PCA system based on split humanized *G. princeps* luciferase (GLuc) used in the present study has been previously described [18]. PCA studies were conducted as already described [22,23]. Briefly, the cells were plated (10,000 cells/well) on white-wall 96-well plates (PerkinElmer Life Sciences) coated with poly-L-lysine (Sigma-Aldrich) and transfected with 125 ng of total DNA per well (62.5 ng of GLuc1 reporter plasmid and 62.5 ng of GLuc2 reporter plasmid) 24 h after plating using JetPei (Polyplus). Luminescence-based detection of PCA signal was done 48 h after transfection with Varioskan Flash plate reader (Thermo Scientific), following well-by-well injection of native coelenterazine (final concentration, 20 µM; Nanolight Technology) as the substrate for the luciferase. Prior the detection, cells were washed with PBS (137 mM NaCl, 2.7 mM KCl, 7.9 mM Na_2_HPO_4_, 1.5 mM KH_2_PO_4_, pH 7.4) and incubated in phenol red-free DMEM (Invitrogen) without serum for 30 min to 1 h. Four replicate wells were used per experimental condition.

### Sucrose density gradient centrifugation

The cells were plated on 10-cm plates and transfected with 9 μg of total DNA per well. Forty-eight hours after transfection, two plates (∼80% confluent) were combined and the cells were washed twice with ice-cold PBS, scraped and extracted on ice for 30 min in a buffer containing 25 mM Tris-HCl, pH 7.4, 150 mM NaCl, 5 mM EDTA, 1 mM PMSF, protease inhibitor cocktail (Roche Molecular Biochemicals), 0.5% (w/v) Lubrol 17A17 (Serva), pH 7.4. Cells were pulled through a 25G needle five times. Protein concentration of extracts was determined by BCA Protein Assay kit (Thermo Scientific) and equal amounts of total protein per sample were used. Protein samples were adjusted to 45% (w/v) sucrose solution (25 mM Tris-HCl, 150 mM NaCl, 5 mM EDTA, pH 7.4) and placed in Ultra-Clear tubes (Beckman). Samples were overlaid with 35% (w/v) and 5% (w/v) sucrose buffer. After centrifugation at 180,000 × *g* for 18 h at 4°C in in the Beckman Coulter Optima MAX XP ultracentrifuge with TLS-55 rotor, 12 fractions were collected from the top of the gradient. SDS was added per each fraction (0.25%, w/v). Fractions were sonicated for 5 min and further analyzed by Western blotting.

### Immunofluorescence microscopy

Immunofluorescence microscopy was done as previously described (Kysenius et al., 2012). Briefly, the cells were plated on glass coverslips coated with poly-L-lysine (Sigma-Aldrich) in 24-well plates and transfected with 1 μg of total DNA per well. Twenty-four hours after transfection, the cells were fixed with 4% (w/v) paraformaldehyde (PFA) in PBS for 20 min and washed with PBS before incubation for 1 h in blocking buffer (1% (w/v) BSA, 0.1% (w/v) gelatin, 5% (v/v) goat or donkey serum, 0.1% (v/v) Triton X-100 and 0.05% (v/v) Tween-20 in PBS). Coverslips were incubated overnight at 4°C with the primary antibodies (HA mouse monoclonal antibody, Sigma-Aldrich) diluted 1:500 in 1% (w/v) BSA, 0.1% (w/v) gelatin and 5% (v/v) serum. The secondary antibodies conjugated with Alexa Fluor (488-goat-anti-mouse; Invitrogen) were diluted 1:2000 in PBS and incubated 1 h at room temperature. Coverslips were finally incubated in Hoechst 33342 (Invitrogen) for nuclear staining before being mounted on microscope slides with Prolong Gold antifade reagent (Invitrogen). Images were acquired with a Zeiss Axio Imager M1 epifluorescence microscope and compiled with Adobe Photoshop.

### Data treatment and statistical analyses

#### Data normalization in LR-PCA

For accurate quantitative analysis, LR-PCA data (luminometric measurements) were normalized to remove both biological and non-biological sources of experimental variability arising from cell culture conditions, inconsistency in procedure and different batch of the luminescent substrate used for an assay. Data in each biological replica were normalized by the sum of measurements of all data points in the replica. For the easier visual comprehension of the graphs, data are expressed as normalized luminescence units (NLU) with median of control being 100 NLU.

#### Statistical analysis

Data from LR-PCA experiments were analyzed with the non-parametric Kruskal–Wallis (K-W) test since the assumptions for parametric tests (normality and homoscedasticity) could not be ensured due to small sample sizes [24]. Significant effects were followed-up with the Dunn’s post-hoc test (in Figure 3A,D,E treatment data points were compared with the control only; in the rest of the figures all data points were compared with each other). All graphs were produced and statistically analyzed in GraphPad Prism. The statistical details of experiments can be found in the corresponding figure legends. *P*-values provided in the figure legends are derived from the K-W test, whereas *P*-values included in the actual figures are derived from post-hoc comparisons. All *P*-values below 0.05 were considered significant. In the figure legends ‘*n*’ represents the number of independent replications of the entity–interventions.

#### Sample size estimation

A sample size calculation for the K-W test was conducted by adjusting the results of a priori power analysis for the corresponding parametric test (one-way ANOVA) by the lowest boundary of the asymptotic relative efficiency (ARE = 0.864). Power analysis revealed that to detect the expected effects size of Cohen’s *f* = 1.4 with power of 0.8 and significance level at 0.05 (two-sided) would require group sizes of *n* = 5 for the 6-group K-W and *n* = 6 for the 3-group K-W (G*Power software) [25,26].

## Results

To develop a sensitive and quantitative assay to monitor trafficking of proteins to membrane rafts in live cells, we utilized a PCA based on the codon-optimized for mammalian expression form of *G. princeps* luciferase (GLuc) [18]. Previously, we have successfully used PCAs based on GLuc for studying cellular regulation of both APP and Tau [22,23,27–30].

We created two reporter constructs, carrying one half of the luciferase protein (either N-terminal 93 amino-acid fragment or C-terminal 76 amino-acid fragment) fused to the ten amino acid-long acylation motif from the Src-family kinase Fyn (referred as LR sequence). This construct serves as a reversible real-time sensor of lipid raft recruitment of proteins carrying a complementary GLuc fragment, generating lipid raft-localization Protein-fragment Complementation Assay (LR-PCA) signal. Acylation motifs from Src-family tyrosine kinases, such as Fyn and Lyn, have been previously demonstrated to successfully target proteins to DRMs and membrane microdomains [20,31–34]. The reporter construct also possesses an HA-tag (residues 98–106 from human influenza hemagglutinin) facilitating immunodetection of the reporter protein. Generated constructs are referred to as LR-GLuc1/HA and LR-GLuc2/HA, where GLuc1 and GLuc2 correspond to N-terminal 93 amino-acid fragment (10.1 kDa) and C-terminal 76 amino-acid fragment (8.2 kDa) of GLuc (excluding 16 amino-acid N-terminal secretion signal sequence), respectively. GLuc1 and GLuc2 were always placed in the C-terminus of the reporter proteins. Figure 1A shows the graphical presentation of LR-GLuc1/HA reporter and the assay principle.

LR-PCA was performed in mouse neuroblastoma N2A cells. Western blot analysis showed that both LR-GLuc1/HA and LR-GLuc2/HA constructs were expressed correctly (Figure 1B). Since the LR-GLuc1/HA reporter construct had higher expression levels than LR-GLuc2/HA (densiometric analysis: 78% vs 22% of total optical intensity), we utilized LR-GLuc1/HA in all LR-PCA experiments that required only one out of two reporters. To exclude the possibility of spontaneous assembly of GLuc1 and GLuc2 fragments, we compared several combinations of reporters with PCA. Co-expression of LR-GLuc1/HA and LR-GLuc2/HA generated a high-level of luminescence signal unlike combinations where at least one PCA reporter did not contain any GLuc reporter protein fragment or targeting motif (Figure 1C). Further, we analyzed how largely non-DRM protein α-secretase ADAM10 (a disintegrin and metalloproteinase domain-containing protein 10) associates with the LR-GLuc1/HA reporter [35]. As interpretation of absolute luminescence values produced by a single interaction is problematic, we compared the level of luminescence generated by the association of ADAM10 reporter (ADAM10-GLuc2) with LR-GLuc1/HA reporter and with three known interacting partners of ADAM10: amyloid precursor-like protein 2 (APLP2), gamma-secretase complex subunit Aph1b, and amyloid precursor protein (APP) [36–39]. Figure 1D demonstrate that co-expression of ADAM10-Gluc2 with LR-GLuc1/HA generated a neglectable level of luminescence signal as compared with the signals generated by its co-expression with APLP2-GLuc2, Aph1b-GLuc2 or APP-GLuc2. Figure 1D also allow to compare the difference in a level of luminescence produced by co-expression of LR-GLuc1/HA reporter with the non-DRM protein ADAM10 and the DRM protein APP.

Next, we tested if the acylation motif from Fyn kinase is sufficient and necessary to recruit the luciferase fragment to DRMs. Therefore, we analyzed GLuc1/HA, lacking the targeting sequence, and LR-GLuc1/HA constructs with Lubrol WX detergent-based density gradient fractionation. Western blot analysis of fractions showed the distribution of the reporters between non-DRM and DRM fractions. The DRM fractions were detected based on the presence of the established DRM marker Flotillin-2, successfully used with Lubrol WX detergent-based fractionation in the N2A cells in a combination with Lubrol WX [40–42]. As shown in Figure 1E,F, GLuc1/HA reporter without the acylation motif was localized exclusively to non-DRM fractions, while the majority of the LR-GLuc1/HA localized to DRM fractions. LR-GLuc2/HA had the same distribution pattern as LR-GLuc1/HA (data not shown).

The raft-targeting motif from the N-terminus of Fyn kinase includes two types of acylation: myristoylation and double palmitoylation; localization to plasma membrane and DRMs requires both modifications [43–45]. Myristoylation is the co-translational static linkage of fatty acid myristate to glycine resides, while palmitoylation is a post-translational reversible linkage of fatty acid palmitate to cysteine residues [46,47]. Thus, in order to confirm that the localization of LR-GLuc1/HA depends on the acylation status of the targeting motif, we mutated key acylation sites in the LR targeting motif: LR(C3,6S)-GLuc1/HA and LR(G2A)-GLuc1/HA [43,44] (Figure 2A). C3,6S mutation prevents palmitoylation but not myristoylation, while G2A mutation prevents both modifications [48]. Both LR(C3,6S)-GLuc1/HA and LR(G2A)-GLuc1/HA constructs were expressed at comparable levels with the wild-type reporter (densiometric analysis: 32% vs 32% vs 36% of total optical intensity) in N2A cells, which allows direct comparison of the PCA signals from these three reporters (Figure 2B). Next, we examined how G2A and C3,6S mutations alter the distribution of the reporter between DRM and non-DRM fractions. Western blot analysis showed that while the majority (63%) of the LR-GLuc1/HA localizes to Flotillin-2 rich fractions (Figure 1F), LR(C3,6S)-GLuc1/HA distributes evenly between non-raft and raft fractions (49% in Flotillin-2 rich fractions), and only minority (13%) of LR(G2A)-GLuc1/HA localizes to Flotillin-2 rich fractions (Figure 2C,D). These data are in agreement with previous studies regarding the role of Fyn N-terminal motif acylation in raft targeting [43,44].

To further assess the functionality and cellular compartmentalization of the LR-PCA reporter and its acylation mutants, we analyzed their subcellular localization by immunofluorescence microscopy. Non-mutated, wild-type LR-GLuc1/HA reporter protein localized mostly to the plasma membrane and to some extent to intracellular vesicles, while LR(C3,6S)-GLuc1/HA localized mostly to intracellular vesicles, and non-acylated LR(G2A)-GLuc1/HA showed diffuse cytosolic and nuclear localization (Figure 2E) [19,45]. These results confirm that acylation effectively drives the GLuc/HA reporter to the plasma membrane.

Finally, we examined if the changes in the localization of the reporter would affect the LR-PCA signal. Therefore, we compared the LR-PCA signal from the non-mutated and mutated reporters with three interacting partners: LR-GLuc2/HA, Fyn-GLuc2 and the β-site amyloid precursor protein cleaving enzyme 1 BACE1-GLuc2 (Figure 2F–H). Figure 2F demonstrates that LR-GLuc2/HA generated high LR-PCA signal with the non-mutated reporter, as previously shown in Figure 1C. LR-GLuc2/HA association with LR(C3,6S)-GLuc1/HA was also rather high, although lower (median 57 NLU, IQR 43–69 NLU) than with the non-mutated LR-PCA reporter (median 100 NLU, IQR 90–120). LR(G2A)-GLuc1/HA reporter association with LR-GLuc2/HA was the lowest (median 23 NLU, IQR 17–23 NLU). These data are in close correspondence with both fractionation and imaging experiments: while LR-GLuc1/HA and LR(C3,6S)-GLuc1/HA localizations considerably overlap, only a minor part of LR(G2A)-GLuc1/HA localized to DRM fractions (Figure 2C–E).

As shown in Figure 2G, LR-PCA data with Fyn-GLuc2 show a similar pattern to that observed in Figure 2F, which is not surprising, since both LR-GLuc2/HA and Fyn-GLuc2 have the same membrane targeting motif. Possible differences in half-live of the reporters and reversibility of the palmitoylation can explain the difference of LR(G2A)-GLuc1/HA LR-PCA signals (Fyn-GLuc2 median 45 NLU, IQR 39–56 NLU; LR-GLuc2/HA median 23 NLU, IQR 17–23 NLU). Fyn and other proteins continuously go through rounds of palmitoylation and depalmitoylation to cycle between the Golgi apparatus and the plasma membrane [49,50]. LR-GLuc1/HA reporter is also expected to undergo the palmitoyl turnover since the core depalmitoylation machinery Acyl-protein thioesterase 1 (APT-1) lacks substrate-specificity, but the estimated palmitoyl turnover rate differs between proteins, which may explain the differences between LR-GLuc2/HA and Fyn-GLuc2 [49].

Next, the LR-PCA reporters were used to study membrane raft localization of BACE1-GLuc2. The association of BACE1-GLuc2 with LR(C3,6S)-GLuc1/HA mutant reporter was at a comparable level (median 88 NLU, IQR 82–94 NLU) to that of the non-mutated reporter (median 100 NLU, IQR 93–105 NLU) (Figure 2G). LR-PCA signal with LR(G2A)-GLuc1/HA was relatively strong as well (median 42 NLU, IQR 35–49 NLU). BACE1 is a membrane-bound aspartyl protease that cleaves various transmembrane proteins [51]. BACE1 is mostly located to late Golgi and to a less extent to endosomes and the plasma membrane, where it also associates with rafts, which agrees with our BACE1-GLuc2 LR-PCA data [52–55].

To demonstrate that LR-PCA can monitor the dynamic localization of a protein of interest to rafts, we examined association of Akt kinase with LR-GLuc1/HA in N2A cells treated with insulin to activate PI3K/Akt pathway, as signaling through this pathway involves dynamic spatial compartmentalization of stimulated Akt to membrane microdomains [56–59]. As expected, the association of Akt with LR-GLuc1/HA increased with insulin stimulation in a dose-dependent manner, with a maximal stimulation at 1 U/ml (median 387 normalized luminescence units [NLU], interquartile range [IQR] 528–243 NLU) in comparison with the control (median 100 NLU, IQR 192–87 NLU) (Figure 3A). This increase in Akt LR/GLuc1/HA association is paralleled by an increase in phospho-Akt(Ser473) level in cells (densiometric analysis, expressed as % change to + Akt-Gluc2/- insulin: 10^−5^ U/ml – 365%, 10^−4^ U/ml – 155%, 10^−3^ U/ml 156%, 10^−2^ U/ml – 181%, 1 U/ml – 175%), indicating activation of the PI3K/Akt signaling pathway in N2A cells (Figure 3B). Insulin stimulation, however, did not alter the phosphorylation of GSK3β at Ser9 to the same extend as for Akt (densiometric analysis, expressed as % change to + Akt-Gluc2/- insulin: 10^−5^ U/ml – 136%, 10^−4^ U/ml – 91%, 10^−3^ U/ml 102%, 10^−2^ U/ml – 137%, 1 U/ml – 109%), suggesting only low inhibitory phosphorylation by Akt [60]. This result is in agreement with the study of Adam et al., which showed that Akt from DRM fractions has low kinase activity toward GSK3β in comparison with Akt from DSM fractions [61].

To demonstrate that LR-PCA is a useful tool in studying dynamic association of disease-associated proteins with rafts and therefore may benefit pharmaceutical studies, we applied our assay to examine APP, whose connection to rafts and cholesterol may contribute in pathogenesis of Alzheimer’s disease (AD) [62,63].

APP undergoes proteolytic processing via either the non-amyloidogenic pathway, which is independent of cholesterol-rich domains, or via the minor amyloidogenic pathway that depends on them [64]. The first cleavage by BACE1 in the amyloidogenic pathway generates a membrane-bound C-terminal fragment (βCTF), which undergoes a further cleavage to produce Aβ peptide, the major constituent of amyloid plaques in AD [65]. A minority of APP associates with membrane microdomains in a dynamic cholesterol-dependent manner [66–68]. Such localization of APP promotes its processing by β-secretase via amyloidogenic pathway [35,64,66]. βCTF almost entirely localizes to DRMs, while the APP intracellular domain (AICD), generated in the subsequent cleavage, does not [69,70]. Therefore, first, we examined if LR-PCA can correctly identify the lipid raft localization of APP and its proteolytic fragments, tagged with a complementary GLuc2 fragment at their C-termini. As expected, membrane-bound APP (median 100 NLU, IQR 80–137 NLU) and βCTF (median 106 NLU, IQR 72–120 NLU), but not the intracellular AICD fragment (median 20 NLU, IQR 11–24 NLU), strongly associated with the LR-GLuc1/HA reporter (Figure 3C).

Since the localization of APP to DRMs and membrane microdomains is cholesterol-dependent, we next investigated whether manipulation of cholesterol level would regulate the association of APP with the LR-GLuc1/HA reporter [66,68]. Thus, we treated cells acutely with methyl-β-cyclodextrin (mβCD) to deplete cholesterol primarily from the plasma membrane [71]. Figure 3D demonstrates that an acute cholesterol depletion decreased the APP association with the GLuc1/HA in a dose-dependent fashion with a maximum effect at 10 mM (10 mM median 55 NLU, IQR 51–59 NLU; control median 100 NLU, IQR 94–103 NLU). In the reverse experiment, we loaded cells with cholesterol using a mβCD/cholesterol complex. Acute cholesterol loading increased the association of APP with GLuc1/HA (100 µM median 130 NLU, IQR 122–145 NLU; control median 100 NLU, IQR 94–103 NLU) (Figure 3E). Collectively, these data show that the LR-PCA is capable of detecting acute changes in membrane domain localization of proteins in living cells.

## Discussion

Here, we have developed and validated a novel live-cell assay for studying raft localization of proteins using a *Gaussia princeps* luciferase-based PCA. We demonstrated that the LR-GLuc1/HA reporter is efficiently targeted to DRMs by two types of acylation in the ten amino-acid long targeting motif from Fyn kinase. The motif and its normal acylation are necessary and sufficient for the reporter localization to DRMs. We also demonstrated that the assay can efficiently detect the dynamic alterations in raft localization of two disease-associated proteins: Akt and APP. Multiple lines of evidence indicate that the alteration in membrane compartmentalization of these proteins contributes to development of pathological conditions, which makes them an exciting target for research [35,56,64,66,72]. PI3K/Akt pathway requires compartmentalization to membrane microdomains for its activation, and misregulation of Akt localization to these microdomains inhibits the pathway and may result in the development of insulin resistance [56,72]. APP localization to rafts, on the contrary, favors the pathological β-secretase processing and the generation of Aβ peptide [35,64,66]. Using LR-PCA, we successfully examined the alteration in membrane localization of Akt in response to insulin and of APP in response to cholesterol level modulation, suggesting that LR-PCA is a reliable assay to study dynamic raft localization.

The LR-PCA is based on *G. princeps* luciferase PCA, which is a very sensitive assay enabling detection of proteins even at low expression levels [18]. The high sensitivity is an essential advantage for LR-PCA since only a minor amount of many disease-associated proteins localizes to lipid rafts. But most importantly, the luciferase complementation is fully reversible, allowing real-time monitoring of dynamic changes in lipid raft localization in intact live cells with a temporal resolution of less than a minute [18]. The main limitation of LR-PCA is that the assay requires overexpression of GLuc fragment-tagged proteins, which may alter the processing, trafficking or the cellular localization of a studied protein [73–77]. The current assay also utilizes transient transfection, which may generate variation between replicas; the generation of stable cell lines is preferable when better control of the overexpression level is required.

While LR-PCA is unable to effectively replace the imaging approaches with their high spatiotemporal resolution, the assay has many advantages over the common simplistic approaches to study protein partitioning to lipid microdomains, such as the density gradient fractionation or the CTxB crosslinking-based conventional microscopy-colocalization measurement. Most importantly, LR-PCA allows quantitative real-time detection of the dynamic partition of protein in the intact plasma membrane with the protein/reporter proximity distance limit lower than that of the ordinary diffraction-limited fluorescence microscope resolution (in the range of 200 nm). Although the precise distance between LR-PCA reporters for *G. princeps* luciferase complementation was not directly assessed, estimations based on the linker size suggest that the 12 amino acid linkers should allow complementation between small reporter fragments such as in LR-PCA at the distance less than 10 nm (≈4 Å per peptide bond × 12 aa × 2 linkers = 96 Å = 9.6 nm) [78]. This distance limit is close to that required for transfer between donor and acceptor fluorophores in Förster Resonance Energy Transfer (FRET) [78,79]. The assay and data processing are rapid and not labor-intensive, requiring only standard molecular biology laboratory equipment (general cell culture equipment, a luminescence plate reader with an injector and a computer), little starting material and no special data analysis software (beside the plate reader data acquisition and statistical analysis software). Therefore, LR-PCA has a potential for low-cost and high-throughput screenings aiming to dissect the mechanism and regulation of protein partitioning to the cholesterol/sphingomyelin rich microdomains.

In conclusion, LR-PCA is a sensitive, dynamic and reliable assay to dissect trafficking mechanisms of various proteins in living cells. The assay is a suitable tool for high-throughput screening approaches, from drug discovery to functional characterization of various disease-associated proteins.

## Supplementary Material

Supplementary Figure S1

## Abbreviations

DRMs: detergent-resistant membranes
DSMs: detergent-soluble membranes
FCS: fluorescence correlation spectroscopy
FRET: Förster Resonance Energy Transfer
GPI: glycophosphatidylinositol
NSOM: near-field scanning optical microscopy
PALM: photoactivated localization microscopy
PCA: Protein-fragment Complementation Assay
PIP_2_: phosphatidyl inositol 4-5-bisphosphate
SIM: structured illumination microscopy
SPT: single particle tracking
STED: stimulated emission depletion
