# Supplementary material for: Live-cell monitoring of protein localization to membrane rafts using protein-fragment complementation
Source: Biosci Rep. 2020 Jan 3;40(1):BSR20191290. doi: 10.1042/BSR20191290 (PMC6944658; doi:10.1042/BSR20191290)
Supplement: Supplementary Figure S1 [file BSR-2019-1290_supp.pdf]

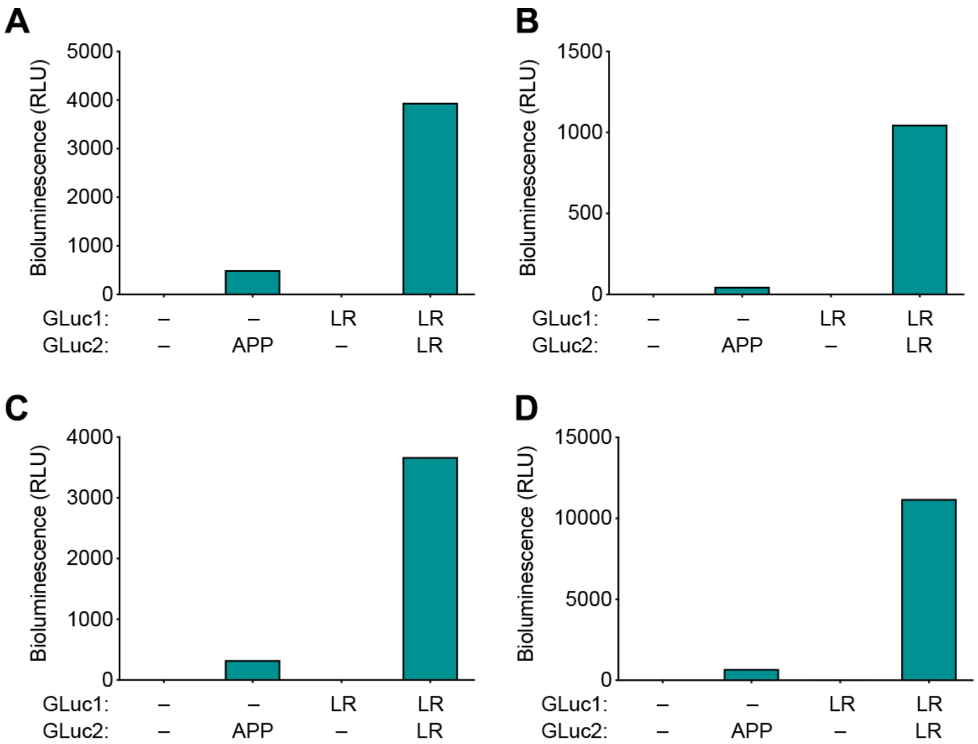

Supplementary figure 1.

Supplementary figure 1. Validation of LR-PCA for detection specificity. N2A cells were transiently transfected with indicated constructs; luminescence signal was measured 48 h post-transfection in live cells. Little or no luminescence is detected when one or both co-expressed plasmids were GLuc1/2 expressing the indicated GLuc fragment alone. The values are bioluminescence signals recorded from expressed pairs of reporter constructs. The panels **A -D** show the results of four individual experiments. The panel **A** was used as the representative experiment in Figure 1C.
